# Influence of evening/night-time birth on maternal/perinatal outcomes in a low-risk population

**DOI:** 10.4274/jtgga.galenos.2020.2020.0081

**Published:** 2020-12-04

**Authors:** Cláudia Rejane Pinheiro Maciel Vidal, Maxsuenia Queiroz Medeiros, Joana Adalgisa Furtado Magalhães Andrade, Edward Araujo Júnior, Francisco Herlânio Costa Carvalho

**Affiliations:** 1 Department of Obstetrics and Gynecology, University of Ceará (UFC), Assis Chateaubriand Maternity School, Fortaleza-CE, Brazil; 2 Department of Obstetrics, Paulista School of Medicine - Federal University of São Paulo (EPM-UNIFESP), São Paulo-SP, Brazil; 3 Medical course, Municipal University of São Caetano do Sul (USCS), São Paulo-SP, Brazil

**Keywords:** Evening/night-time, labor, delivery, adverse maternal/perinatal outcomes

## Abstract

**Objective:**

To compare maternal and perinatal outcomes between day-time and evening/night-time births in a low-risk population.

**Material and Methods:**

The present study had a retrospective and cross-sectional design. The study recruited 421 pregnant women admitted for spontaneous or induced labor, with singleton, full-term pregnancy, without comorbidities, and with birthweight between 2,500 and 4,499 g. Maternal data, including severe bleeding, need for blood transfusion, puerperal infection, and admission to the intensive care unit, and neonatal data including birthweight, Apgar scores at first and fifth minute, oxygen administration, resuscitation, admission to the neonatal care unit, infection, and blood transfusion, were evaluated. Univariate and multivariate analysis and calculation of the prevalence ratio (PR) were performed with a 95% confidence interval (CI).

**Results:**

There were no differences in factors of maternal morbidity between delivery times. Newborns delivered during the evening/night-time had a higher prevalence of infection (15.3% vs 7.9%, p=0.019, PR: 2.11, CI 95% 1.13-3.93) and hospitalization in the neonatal care unit (25.8% vs 10.4%, p<0.001, PR: 2.99, CI 95% 1.76-5.10). There was no difference in other perinatal morbidities examined.

**Conclusion:**

Evening/night-time births were associated with a higher prevalence of infection and the need for admission to an intensive care unit.

## Introduction

In Brazil, and worldwide, the emphasis on humanized childbirth care has increased. In this model of humanized childbirth, several measurements are adopted to improve access, reception, quality, and resolution of obstetric care; actions that are important to reduce maternal and perinatal morbidity (1).

The Brazilian Ministry of Health, which aims to provide better maternal and perinatal outcomes, has created public policies that seek to improve the quality of maternal and child healthcare. Despite expanding prenatal care, delivery, and newborn care, maternal and perinatal mortality rates are still high, as evidenced by intense hospitalization and medicalization of childbirth (2,3).

A higher risk of adverse perinatal outcomes among parturients admitted during off-hours (weekends, evening, during the night) compared to office hours, has led to questions about the quality of care provided during off-hours (4,5). According to de Graaf et al. (6), these findings are evidence of organizational problems that directly affect perinatal outcomes. According to Gijsen et al. (7) evening and nighttime deliveries are associated with a higher risk of adverse perinatal outcomes when compared with daytime deliveries. These risks are concentrated in subgroups that involve induction or augmentation of labor or emergency cesarean section.

In Brazil, no studies have analyzed the childbirth care received by parturients in maternity hospitals during the day, in the evening, or at night. The study objective was to compare maternal and perinatal outcomes between daytime and nighttime births in a low-risk population in a tertiary healthcare setting.

## Material and Methods

The present study was a cross-sectional study with a retrospective design. Data was collected from medical records and birth notification forms from April 1^st^, 2014 to March 31^st^, 2015. The research complied with resolution no 466/2012 of the National Health Council and was approved by the local ethics committee (approval number: 957,050).

To compose the sample, a finite size calculation was performed with a 95% confidence interval (CI) and a maximum error of 5%, resulting in a sample size of 351 based on the number of deliveries in the period of one year, between 2014 and 2015 with 4,099 deliveries performed. In addition, 20% was added to cover participant drop-out, thus resulting in a final recruitment target of 421 participants.

The study included parturients admitted to the obstetric center during spontaneous or induced labor with a live fetus upon admission, and with singleton, full-term pregnancy and without comorbidities, which included a history of diabetes and/or chronic or gestational arterial hypertension, human immunodeficiency virus infection, and/or positive venereal diseases research laboratory. Fetal birth weights should be between 2,500 g and 4,499 g. Parturients admitted for elective cesarean section were excluded. For the purpose of the study daytime hours were defined as 7.00 a.m. to 6.59 p.m. and evening/nighttime hours as 7.00 p.m. to 6.59 a.m. This division was due to standardization of staff shift patterns. Mother and baby pairs were divided into two groups based on delivery falling into one of these two time groupings.

**Figure 1:**
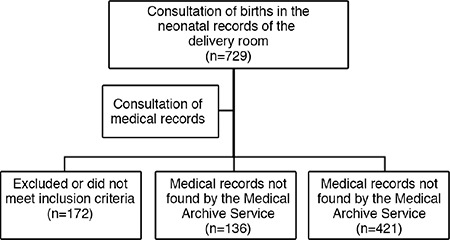
Recruitment of the participants

Initially, a list of 729 patient names was constructed. The list included patients who had given birth in that period following examination of neonatology records. After further examination of patients’ medical records, 172 women were removed because they did not meet the inclusion criteria. A total of 136 medical records were requested but were not available. Therefore, the final sample consisted of 421 women and their newborns, since there were no fetal or neonatal deaths (Figure 1).

Epidemiological and obstetric care data were collected. Data items collected were in four basic categories. These were: i) sociodemographic characteristics including race, age, marital status and education; ii) obstetric clinical characteristics including number of pregnancies (including the current pregnancy), number of previous cesarean sections, number of prenatal consultations, Robson’s classification, type of delivery, professional who attended the delivery; iii) maternal morbidity including severe bleeding, need for blood transfusion, puerperal infection, and admission to the intensive unit care (ICU); and iv) neonatal morbidity including Apgar scores at 1^st^ and 5^th^ minute, need for oxygen, resuscitation in the delivery room, admission to the neonatal ICU, neonatal jaundice, history of infection and blood transfusion.

### Statistical analysis

The data was analyzed using Stata^®^ 11.2 software (Stata Corp., College Station, TX, USA). Univariate analysis was performed, with the calculation of proportions for categorical variables and measurements of central tendency for numerical variables. The differences between the study groups were assessed for statistical significance using the chi-squared test or Fisher’s exact tests for categorical variables. A significance level of p<0.05 was considered statistically significant. The prevalence ratio (PR) was calculated with a 95% CI.

## Results

Table 1 shows the comparison of sociodemographic and obstetric characteristics among the participants according to the two birth times. Of the 421 deliveries, 190 (45.1%) occurred during the evening/night-time, while 231 (54.9%) occurred during the day-time. There was a predominance of patients who were non-white and a higher proportion of women aged 20-35 years (63.6%). Regarding relationship status, 74.7% of patients had a partner. In educational terms, 56.3% had attended high school, while 54.2% of participants were primiparous. The majority of patients (64.5%) had at least six prenatal consultations and had not previously undergone cesarean section (93.7%) (Table 1).

When the parturients were separated by Robson’s classification, there was no statistical difference between the period of delivery. 99.1% of the women involved in the study were classified in the first five groups of the Robson classification, 0.9% in groups 6 and 7 and none of them in the groups 8, 9 and 10.

Table 2 shows that during the evening and at night, maternal morbidity was more prevalent, but was not significantly different when compared with that observed during the daytime. Maternal morbidity was low in both periods considered.

The mean labor duration was longer for cesarean section deliveries (13.8 hours) than the mean labor duration of vaginal deliveries (8.34 hours). The prevalence of cesarean section was not different between daytime and evening/nighttime births. Among the cesarean sections, the main indications were fetal distress (29%), cephalopelvic disproportion (28%), functional dystocia (22%), and ≥2 previous cesarean sections (19%).

Table 3 shows the perinatal outcomes. Neonatal infection was more frequent in deliveries that took place in the evening and during the night (PR: 2.11). There was no difference in Apgar scores, need for oxygen, need for resuscitation, or jaundice. A greater number of hospitalizations in the neonatal ICU (PR: 2.99) were observed during the evening and at nighttime.

Multivariate analysis did not show any statistical difference between the analyzed data. Infection and admission at ICU were the factors that showed association with the delivery shift in the univariate analysis. Comparison between the two groups for the variable “number of previous cesarean sections” resulted in a p<0.2, which could suggest some interaction with the delivery type. However on univariate analysis history of previous cesarean section was shown to have had no influence on the results (Table 4).

Multivariate analysis was performed for outcomes that were shown to be statistically different between the shifts in which the birth occurred. Multivariate analyses for the outcomes of infection, admission of the newborn, type of delivery and presence of previous cesarean section are shown in Table 4, 5. When performing the analysis, the outcome of infection and admission of the newborn did not show statistically significant association, and neither did the delivery shift or previous cesarean section.

## Discussion

It is encouraging that among the main results found, there was an absence of difference in maternal morbidity between delivery times. While newborns at evening/night-time had a higher prevalence of infection and hospitalization in the ICU, there was no significance differences for other perinatal morbidities.

The difficulty of accessing the patients’ medical records, due to logistical issues and the retrospective design, together with data being collected from secondary sources are limitations of the study.

Evening and night-time deliveries are associated with increased perinatal mortality and adverse perinatal outcomes. Gijsen et al. (7) observed that there was a higher risk of adverse perinatal outcomes among evening/night-time deliveries, regardless of whether the pregnant woman was in the hospital before the delivery or she was referred during labor, and proposed that newborns who were delivered during the evening and night-time may have been exposed to a longer first phase of labor and were thus at higher risk of adverse perinatal outcomes. These results can be compared to the current study which showed lower Apgar scores at the 1^st^ and 5^th^ minute, a higher prevalence of newborns admitted to the neonatal ICU and infection when delivered during the evening/night-time. Hospitalization in the ICU was likely related to the higher risk of infection presented by these newborns. No significant difference was observed regarding the need to use oxygen and resuscitation in the delivery room when comparing birth shifts.

In the present study, evening/night-time deliveries were associated with puerperal infection, hysterectomy and need for admission to the ICU. Lyndon et al. (8) corroborate these findings, since they identified that severe morbidity from heart failure, severe postpartum hemorrhage, and sepsis were associated with evening and nighttime deliveries. These authors demonstrated that evening and nighttime delivery is a risk factor for maternal morbidity, regardless of other obstetric and sociodemographic risk factors, such as cesarean section, race, and education (8).

Bell et al. (9) analyzed the impact of birth during the evening/night-time in the context of maternal or perinatal morbidity and mortality and observed that pregnant women who gave birth during the evening or at night-time were less likely to have hypertension or pre-eclampsia and were less likely to require cesarean section delivery. Conversely, a higher percentage of women who gave birth at the end of the night presented with antepartum hemorrhage and evening and night-time deliveries were associated with lower Apgar scores.

Mgaya et al. (10) observed that deliveries during the evening/night-time were associated with a higher proportion of adverse perinatal outcomes, including low Apgar scores, fetal distress, early and neonatal death, and stillborn cases. We did not register any maternal or neonatal deaths during the different delivery times. Births at night, weekends and holidays are associated with a higher rate of unfavorable umbilical artery pH indices, with pH values <7.10 and Apgar scores <5 at 5 minutes (11). The objective of this study was not to evaluate the pH results of umbilical cord blood, as it is not routine in our service. However such data is an important indicator of the condition of the fetus at birth.

Aiken et al. (12) reported no differences in the risk of adverse perinatal outcomes between deliveries that were carried out during the day and those that were carried out in the evening or at night. However, they suggested that the number of hours already worked by clinical staff before providing assistance for unscheduled deliveries significantly influenced the risk of adverse perinatal outcomes. In an Irish study in 2014, deliveries at a tertiary obstetric unit that occurred at night were associated with a potential increase in rates of adverse maternal and neonatal outcomes. However, they did not identify a difference in birth weight in babies during the two periods, and when examining the mode of delivery, they found that women who gave birth at night were less likely to do so by caesarean section. Hehir et al. (13) in 2014 suggested that maternal and neonatal complications may occur at night due to the reduced number of staff and, in keeping with the report of Aiken et al. (12), fatigue, which may interfere with decision making and management of patients. Fatigue generated by long hours of work before taking a shift in a maternity ward, excessive hours of work, and excessive responsibility for parturients generated by the reduced number of health professionals, especially at night-time, seem to interfere in decision-making and quality of care provided, relating to possible adverse maternal and perinatal outcomes. Policy makers should ensure there is adequate financial and systemic support for the allocation of human resources and increase the provision of labor facilities in vulnerable areas, in addition to increasing staff numbers during the weekends or night-time to improve the quality of maternal intra- and post-delivery care (14).

The institution where the research was undertaken is considered a reference for good practices in care and birth, in addition to receiving higher risk patients from all municipalities in the state. This results in a large number of current births, on average 4,100 annually, suggesting that the findings can be extrapolated to the local population, although it should be borne in mind that a higher proportion of difficult deliveries is likely in the current cohort.

The findings of this study demonstrate that births occurring during the night shift were related to adverse neonatal outcomes. This data demonstrates the need for institutional assistance for the newborn and, perhaps, a need for an assessment of the performance of existing routines. There is the possibility that these results are replicated in other institutions, and it is important to highlight the necessary care standards regardless of the time of the assistance provided. These results also highlight the importance of training so that standardized practices are available. We believe that there should be further studies, which may identify factors affecting the quality of the assistance provided, in order to optimize delivery assistance and which would result in excellent service provision, with the least possible error.

The present study did not receive external funding and the authors declare that there is no conflict of interest that could constitute an impediment to the publication of this article.

## Conclusion

Evening/night-time births were not associated with adverse maternal outcomes. However, a higher prevalence of adverse neonatal outcomes, in particular infection and requirement for ICU admission, were found in neonates delivered in the evening/night-time period.

## Figures and Tables

**Table 1:**
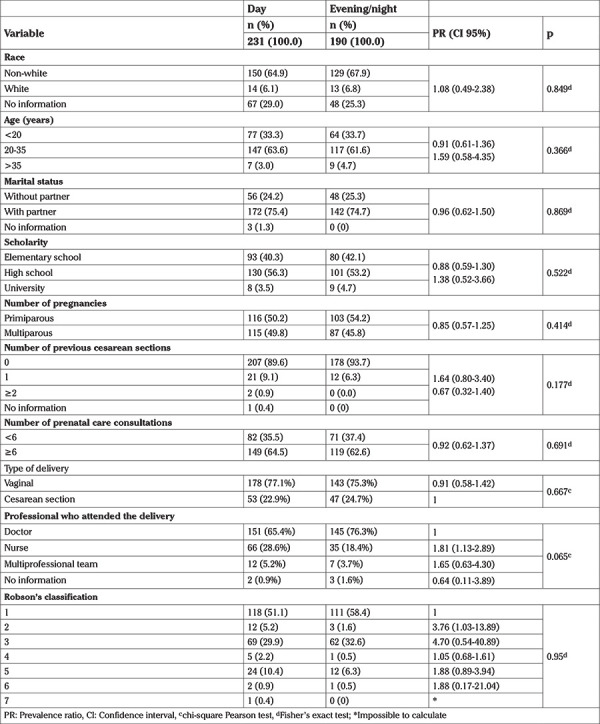
Sociodemographic and obstetric characteristics according to the birth time

**Table 2:**
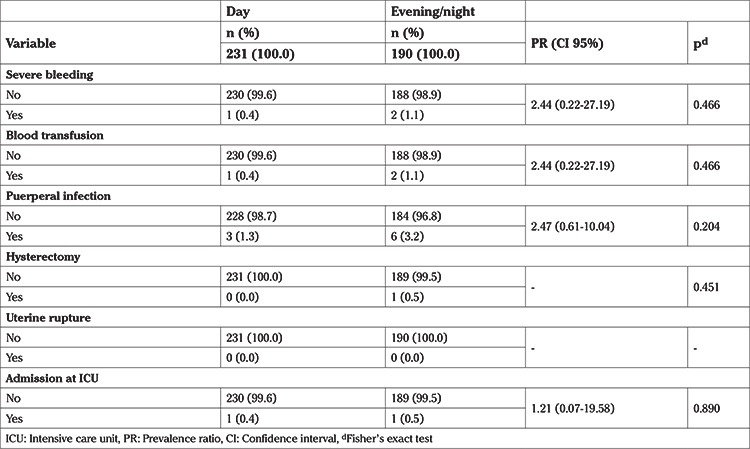
Maternal morbidity according to the birth time

**Table 3:**
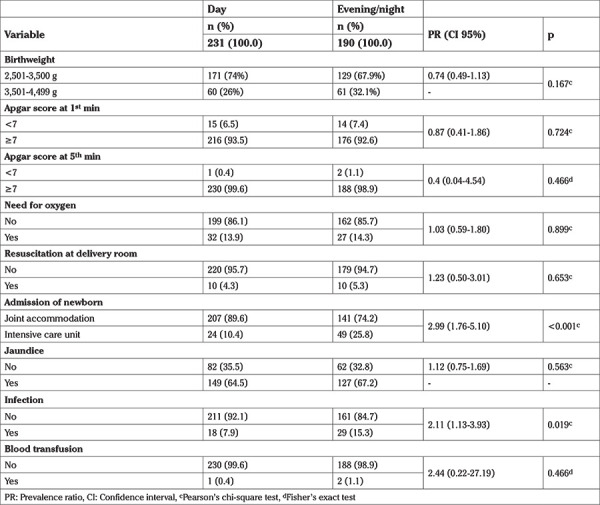
Perinatal outcomes according to the birth time

**Table 4:**
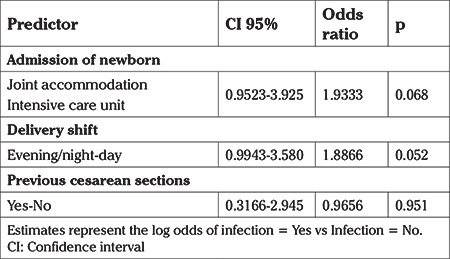
Multivariate analysis

**Table 5:**
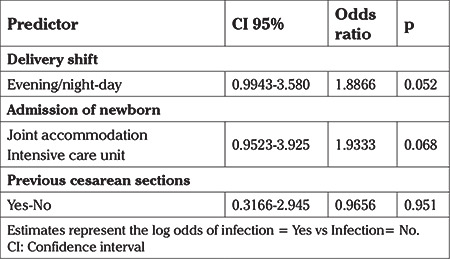
Multivariate analysis
